# CCR3 plays a role in murine age-related cognitive changes and T-cell infiltration into the brain

**DOI:** 10.1038/s42003-023-04665-w

**Published:** 2023-03-18

**Authors:** Sanket V. Rege, Arnaud Teichert, Juliet Masumi, Onkar S. Dhande, Reema Harish, Brett W. Higgins, Yesenia Lopez, Lily Akrapongpisak, Hannah Hackbart, Sofia Caryotakis, Dino P. Leone, Balazs Szoke, Jonas Hannestad, Karoly Nikolich, Steven P. Braithwaite, S. Sakura Minami

**Affiliations:** Alkahest, Inc., San Carlos, CA USA

**Keywords:** Neuroimmunology, Cognitive ageing

## Abstract

Targeting immune-mediated, age-related, biology has the potential to be a transformative therapeutic strategy. However, the redundant nature of the multiple cytokines that change with aging requires identification of a master downstream regulator to successfully exert therapeutic efficacy. Here, we discovered CCR3 as a prime candidate, and inhibition of CCR3 has pro-cognitive benefits in mice, but these benefits are not driven by an obvious direct action on central nervous system (CNS)-resident cells. Instead, CCR3-expressing T cells in the periphery that are modulated in aging inhibit infiltration of these T cells across the blood-brain barrier and reduce neuroinflammation. The axis of CCR3-expressing T cells influencing crosstalk from periphery to brain provides a therapeutically tractable link. These findings indicate the broad therapeutic potential of CCR3 inhibition in a spectrum of neuroinflammatory diseases of aging.

## Abstract (web summary)

CCR3 is shown to play a role in age-related T cell infiltration into the brain in mice as well as age-related changes in cognition, highlighting its therapeutic potential for age-related diseases.

## Introduction

Aging is characterized by changes in both innate and adaptive immune function, including a progressive state of chronic low-grade inflammation, or inflammaging. These immune changes can lead to the development and/or progression of a multitude of age-related diseases, including neurodegenerative diseases such as Alzheimer’s and Parkinson’s diseases, the two most prevalent neurodegenerative disorders affecting the elderly. Development of new therapeutic strategies targeting these age-related processes themselves presents a possible avenue to maintain quality of life in aging.

Inflammaging is characterized by elevations in plasma and tissue levels of specific immune mediators (e.g., proinflammatory cytokines), shifts in immune cell ratios and function, and increased numbers and activation state of microglia in the brain^1,2^. Many of these inflammatory markers that are increased in the circulation with age are known drivers of dysfunction and can impact diverse biological processes that comprise aging^3,4^. CCL11 (Eotaxin) is one such factor that is increased in the plasma and brain of aged mice and humans^5–7^. Recombinant CCL11 administered to young mice induces cognitive impairment^8^, suggesting that individual immune factors can drive aging-associated behavior deficits. However, it is likely that a combination of factors that increase systemically contribute to this process. For instance, other chemokines upregulated with aging and disease, such as CCL5 (RANTES) or CCL3 (MIP-1α), can also negatively impact cognitive and motor function^9,10^. These multiple parallel and divergent changes may partially account for why therapeutic targeting of inflammatory processes has thus far been unsuccessful in disorders such as Alzheimer’s disease^11,12^ and highlight the importance of identifying and targeting critical hubs that are responsible for multiple disease pathways in parallel.

Furthermore, whether the inflammatory changes detected in the circulation are directly related to effects observed in specific organ systems, especially those that are immune privileged such as the brain, remains unknown. Although it is understood that peripheral inflammation can affect the brain, it is not clear whether chronic brain inflammaging originates in the periphery or whether the aging brain is associated with local brain inflammation that occurs in parallel with, but independently of, systemic inflammaging. Various lines of evidence suggest that the two processes (systemic and central inflammaging) are related and interdependent^13,14^. For example, experimentally induced systemic inflammation (e.g., endotoxin administration) has effects on the brain in both rodents and primates^15,16^. These effects may be mediated by direct signaling through vagal afferents, through cytokines in the circulation that signal across the blood-brain barrier, or by immune cells infiltrating the brain. Some cytokines that increase with experimental systemic inflammation also increase with age, providing a common pathway to interrogate aging-associated inflammation. Here, we sought to identify critical hubs for these aging-related inflammatory changes and determine how they influence brain and cognitive function, in order to overcome the challenges of targeting inflammaging. Understanding critical targets and underlying inflammatory mechanisms can lead to powerful therapeutic approaches.

## Results

### CCR3 as a convergent target in aging

**Fig. 1:**
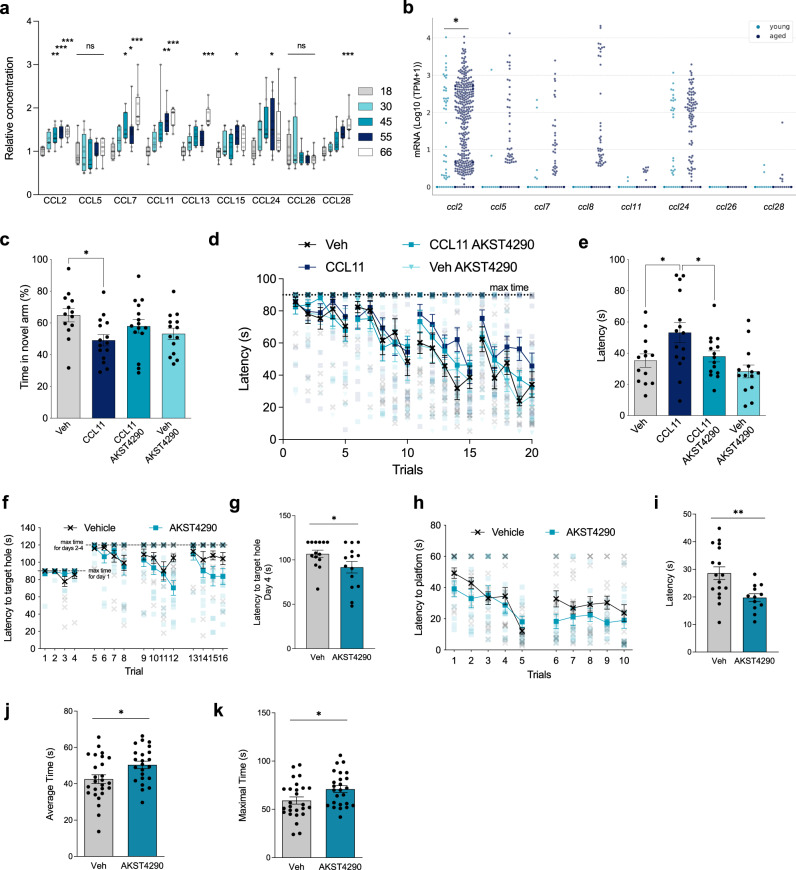
Plasma chemokines increase with aging, with impact on memory and motor function. **a** CCR3 ligands measured from pooled plasma samples from individuals in different age groups (*n* = 8; *p < 0.05, **p < 0.01, ***p < 0.001, ****p < 0.0001; Kruskal Wallis with Dunn Test). **b** Single cell RNA-seq expression analysis of CCR3 ligands in microglia from young and old C57Bl/6 dentate gyrus. Swarm plots show expression values in transcripts per million (TPM) for each gene for cells from young (light blue) and aged (dark blue) animals. Each dot represents the expression value of the respective gene in one cell (*p < 0.05; Benjamini–Hochberg corrected; MAST package, see methods). **c**–**e** Behavioral analyses conducted on 2-month-old C57Bl/6 mice treated with vehicle (Veh) (*n* = 11), recombinant CCL11 i.p. (*n* = 15), recombinant CCL11 i.p. with AKST4290 p.o. (*n* = 15), and AKST4290 p.o. (14). **c** Percent time spent in novel arm in the Y-Maze test (*n* = 11, 15, 15, 14); *p < 0.05; one-way ANOVA and Kruskal–Wallis test). **d** Latency time in seconds to find the escape hole on all days of testing in Barnes Maze (*n* = 11, 15, 15, 14; ****p < 0.0001; Marginal Survival Analysis and Cox Proportional Hazards). **e** Average latency time in seconds to find the escape hole over the last 3 trials on the final day of testing (*n* = 11, 15, 15, 14; *p < 0.05; Mann–Whitney test). **f**–**k** Behavioral analyses conducted on 21-month-old C57Bl/6 mice treated with vehicle or AKST4290 for 5 weeks. **f** Time taken to find the target hole on each day in the Barnes Maze test (*n* = 14; ****p < 0.0001; Marginal Survival Analysis and Cox Proportional Hazards). **g** Average latency to find target hole on last day of Barnes Maze test (*n* = 14; *p < 0.05; Mann–Whitney test). **h** Time taken to find platform for each trial over 2 days in the RAWM test (*n* = 17, 12). **i** Average latency to find the platform for each day in the RAWM test, from an average of 5 trials each day (*n* = 17, 12; **p < 0.01; Mann–Whitney test). **j** Average time mice were able to stay on the rotarod over the 3 trials during the testing phase of Rotarod (*n* = 26, 23; *p < 0.05; unpaired *t*-test). **k** Maximum time mice were able to stay on across the 3 trials during the testing phase of Rotarod (*n* = 26, 25; *p < 0.05; unpaired *t*-test;). All data shown are mean ± standard error of the mean.

Although it is known that inflammaging is linked to a broad and complex spectrum of chemokines and their receptors, key hubs may be identified as master regulators of these cascades. Of the chemokine receptor family, CCR3 clearly stands out as one of these potential hubs, as it is expressed on many cell types, including eosinophils, basophils, T cells, and monocytes, and binds many more chemokine ligands than other members of the chemokine receptor family^17–19^. CCR3 is involved in a variety of functions including cell migration, allergic response, and parasitic infection^20–22^. Thus, we undertook a proteomic approach to determine which of these interactions were changed with age in human plasma. Of the nine CCR3 ligands measured, seven were significantly upregulated with age - CCL2, CCL7, CCL11, CCL13, CCL15, CCL24, and CCL28 (Fig. 1a). Transcript levels for many of these CCR3 ligands were also increased in the aging mouse brain, especially within microglia in the dentate gyrus, as detected by single cell transcriptomics (*ccl13* and *ccl15* expressed in humans, but not in mice) (Fig. 1b, Supplementary Fig. 1). Among the chemokines, *ccl2* had the greatest level of expression in microglia from young mice, and this significantly increased further with age (Fig. 1b). Very few cells expressed *ccl5, ccl7, ccl8, ccl24*, and *ccl28* in the young brain; however, aging enhanced the expression of all. These data from plasma and brain raise the possibility of a heightened role for CCR3 in aging and are in line with recent findings of an increase in expression of *ccl2* and *ccl5* within microglia of the subventricular zone with aging, where robust T-cell infiltration was observed^23^. CCR3, as a common receptor for these ligands, may be a central effector of aging biology; thus, we postulated that inhibition of CCR3 would improve aging-associated deficits.

### CCR3 inhibition improves cognitive and motor function

Using CCL11, the primary ligand of CCR3, which increases with age in the plasma and brain of mice and humans^5–7^, we confirmed that it is a driver of cognitive dysfunction when administered peripherally to young mice (Fig. 1c–e)^8^. To further assess a critical role for CCR3, we tested CCL24, another ligand which increases with age, and found that administration of recombinant CCL24 also induced cognitive deficits (Supplementary Fig. 2). Due to this key role, we hypothesized that CCR3 inhibition could prevent chemokine-induced behavioral deficits. 9-week-old mice were treated with recombinant mouse CCL11 systemically for 3 weeks with concurrent administration of AKST4290, a selective, potent, orally bioavailable, small molecule inhibitor of CCR3 with no significant activity against 80 other receptors, ion channels and transporters (Supplemental Table 1). CCL11-induced impairments in learning and memory in the Barnes maze were significantly improved with AKST4290 treatment, however there was no significant improvement observed in the Y-maze test (Fig. 1c–e). Young mice treated with AKST4290 alone did not exhibit any deficits in learning and did not perform differently from vehicle-treated mice. Extending this finding to animals that have endogenous aging-associated increases in CCL11, 21-month-old mice which naturally demonstrate impaired motor and cognitive function were treated with AKST4290 for 5 weeks. Inhibition of CCR3 by AKST4290 significantly improved learning and memory in the Barnes maze and radial arm water maze (RAWM) and improved motor function in the rotarod test in aged mice (Fig. 1f–k). Taken together, these data suggest that systemic elevation in CCL11, both acutely by administration in young mice or chronically due to inflammaging in aged mice, results in cognitive dysfunction that can be rescued by CCR3 inhibition.

### Brain penetrance of CCR3 inhibitor

**Fig. 2:**
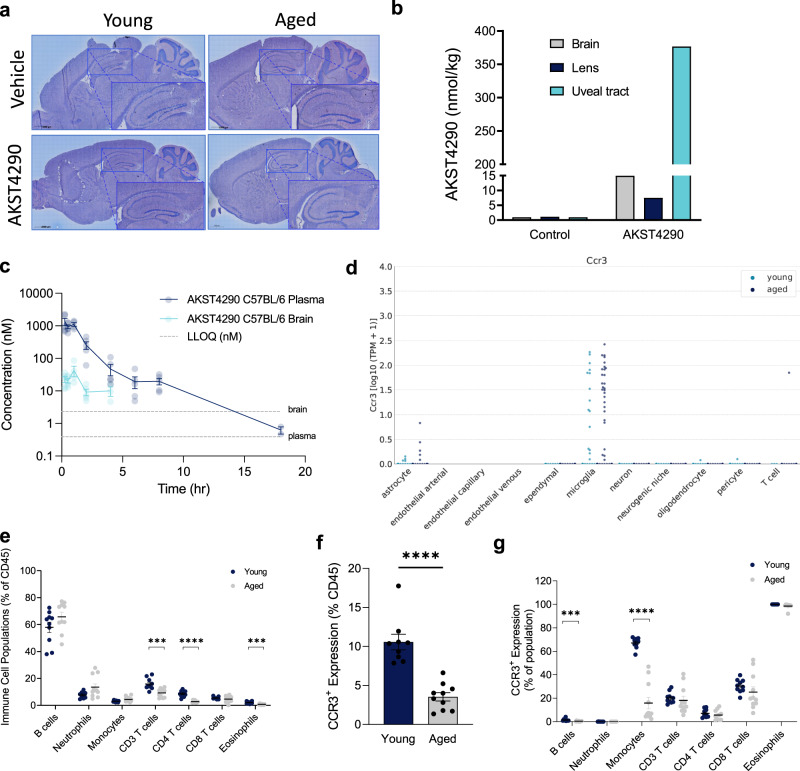
CCR3 is primarily expressed in the periphery and is modulated in aging. **a** Representative images from microautoradiography (MARG) analysis of brain tissue 2 h following treatment with 30 mg/kg [^14^C]-AKST4290 p.o. **b** Quantification by autoradiography (ARG) of various tissues in an aged mouse 2 h following treatment with 30 mg/kg [^14^C]-AKST4290 p.o. **c** AKST4290 levels measured via liquid chromatography-tandem mass spectrometry (LC-MS/MS) in the plasma and brain cortical tissue 2 h after a 30 mg/kg dose p.o. (*n* = 5, 5, 2). **d** Single cell RNA-seq expression analysis of CCR3 expression among major cell types in cells (transcripts per million) from young (light blue) and old (dark blue) C57Bl/6 dentate gyrus. Significant expression of CCR3 is detected in a subset of microglia cells from aged animals. **e**–**g** Flow cytometric analysis of surface markers in whole blood cells from 9-week-old and 26-month-old C57Bl/6 mice. Whole blood cells were stained for markers of interest on eosinophils (SiglecF^+^), monocytes (CD45^+^B220^-^Ly6G^-^CD3^-^CD170^-^SSC_low_), T cells (CD3^+^), T helper cells (CD4^+^), cytotoxic T cells (CD8^+^), B cells (B220^+^), and neutrophils (Ly6G^+^). **e** Respective frequencies of cell populations were normalized to total CD45^+^ cells (*p < 0.05, unpaired *t*-test, *n* = 10, 11). **f**, **g** Flow cytometric analysis of CCR3 expression was shown as percent of total CD45^+^ cells (**f**) as well as percent of the parent population (**g**) with CCR3^+^ signal on immune cell populations (*p < 0.05, **p < 0.01, ***p < 0.001, ****p < 0.0001, unpaired *t-*test, *n* = 9, 10). All data shown are mean ± standard error of the mean.

The most plausible mechanism of action for AKST4290 resulting in the observed CNS effects would be a direct effect on cells in the CNS expressing CCR3. In fact, previous studies conducted in APP/PS1 mice, which show elevated CCR3 levels in the brain^24^, showed improvement in learning and memory with a brain penetrant CCR3 inhibitor^25^. However, while AKST4290 has a favorable lipophilicity for brain penetrance (miLogP 2.19), the total polar surface area and its high molecular weight are predicted to impair brain penetrance. We tested experimentally for brain-specific penetrance using microautoradiography (MARG) in young and aged mice dosed with [^14^C]-AKST4290 (Fig. 2a); no compound was detected in the brain. Quantification by autoradiography (ARG) in aged mice showed marginal levels of drug in the brain and lens but significant levels in the uveal tract (Fig. 2b). Mass spectrometry-based analysis of AKST4290 in the plasma and brain tissue of mice dosed with AKST4290 also demonstrated an acute transient increase in the brain that was quickly reduced by 2 h post-dose to levels lower than that required for pharmacological activity (Fig. 2c, Supplementary Fig. 3). Therefore, this raised the intriguing possibility that an alternative, periphery-driven pathway exists for CCR3 effects in the CNS.

### CCR3 localization in brain and blood

In parallel, we examined the localization of CCR3 in the mouse brain using single cell RNA-seq technology (Fig. 2d). Since *ccr3* expression may be altered in the presence of certain neuroinflammatory mediators or in the context of aging or disease^20,22,26–28^, we examined the brains of both young 9-week-old and aged 21-month-old mice. Of all brain cells, only a few microglia expressed *ccr3* in either young or aged mice, with no apparent age-dependency and no expression on neurons detected. These findings are in agreement with other single cell transcriptomic studies^23,29^, but are in contradiction with prior immunohistochemical reports^29^. These data on drug and receptor distribution strongly suggest that the site of action for CCR3 inhibition which results in CNS efficacy is in the periphery. CCR3 is predominantly expressed on basophils and eosinophils, with some expression on Th2 lymphocytes and myeloid cells^30,31^; however, the specificity of expression varies according to the model and the assay used^32–34^. We therefore considered whether immune cell populations were altered in the context of aging (Fig. 2e–g, Supplementary Fig. 4). Interestingly, the proportion of eosinophils and T cells decreases significantly in the blood with age, raising the possibility that these cells may be depleted in the periphery due to increased recruitment to inflamed tissues (Fig. 2e). When assessing the total number of CCR3-expressing cells in blood, there was a significant decrease with aging (Fig. 2f), raising the possibility that CCR3 could be mediating the trafficking of these cells from the circulation to tissue sites of injury. Upon investigation of specific cell subsets, nearly all eosinophils express CCR3 in both young and aged mice as expected; however, there was a significant proportion of monocytes and T cells which also expressed CCR3 in young mice. In aged mice, the proportion of CCR3-expressing monocytes decreased, resulting in T cells and monocytes contributing similarly to the population of circulating CCR3-expressing cells with age (Fig. 2g). This raises the possibility that CCR3 may play a larger role in non-eosinophil biology, specifically that of monocytes and T cells partitioning between blood and tissues, than previously thought.

### CCR3 inhibition reduces T-cell infiltration in aging

**Fig. 3:**
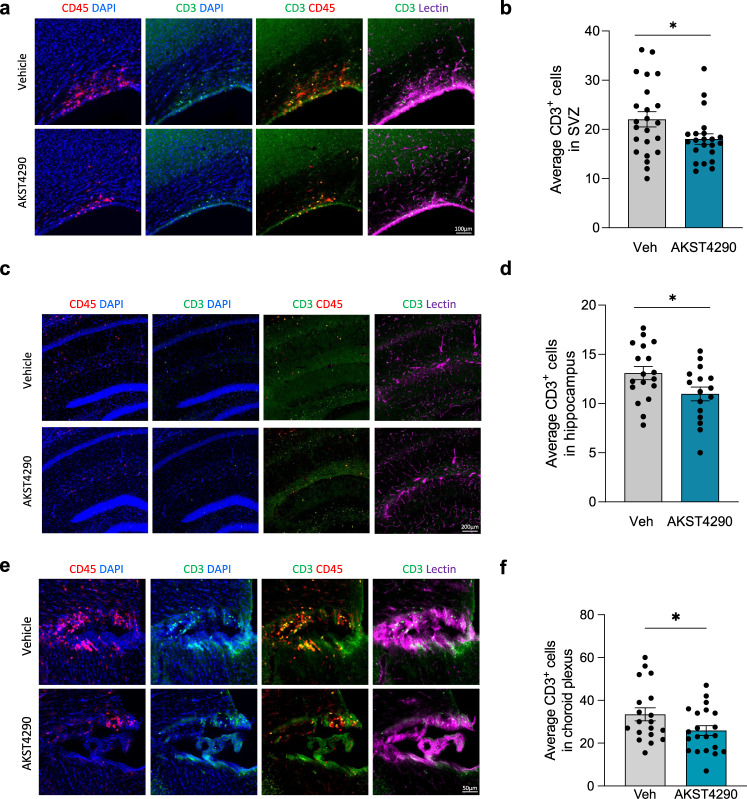
T-cell infiltration in the aged mouse brain is reduced by CCR3 inhibition. 24-month-old male mice were treated with vehicle or AKST4290 for 5 weeks. **a** Representative images of CD3 T cells co-stained with CD45 and vessel marker Lectin in the subventricular zone (SVZ). **b** Average number of CD3^+^ T cells in the SVZ (*p < 0.05, unpaired *t-*test; *n* = 23, 22). **c** Representative images of CD3^+^ T cells co-stained with CD45^+^ macrophages and vessel marker Lectin in the hippocampus. **d** Average number of CD3^+^ T cells in the hippocampus (*p < 0.05, unpaired *t-*test; *n* = 18, 16). **e** Representative images of CD3^+^ T cells co-stained with CD45^+^ macrophages and vessel marker Lectin in the choroid plexus of the third ventricle. **f** Average number of CD3^+^ T cells in the choroid plexus (*p < 0.05, unpaired *t*-test; *n* = 21, 20). All data shown are mean ± standard error of the mean.

The significant numbers of CCR3-expressing T cells and monocytes in the blood of young animals, the changes observed with aging (Fig. 2e–g), coupled with the limited brain penetrance of AKST4290, suggest that the effects of AKST4290 may be mediated by a peripheral immune cell population expressing CCR3. We therefore first determined whether aging-associated T-cell infiltration into the brain was impacted by CCR3 inhibition. 24-month-old mice were treated with AKST4290 for 5 weeks and the numbers of infiltrating T cells in multiple brain regions were quantified (Fig. 3). AKST4290 treatment significantly reduced the numbers of infiltrating CD3^+^ T cells in the subventricular zone (SVZ), hippocampus, and choroid plexus, a known gateway for peripheral immune cells^34^. Furthermore, CD3^+^ T cells were found both in the parenchyma and in the blood vessels (stained with lectin) of the brain, indicating both increased infiltration inside the tissue and increased recruitment to the brain.

**Fig. 4:**
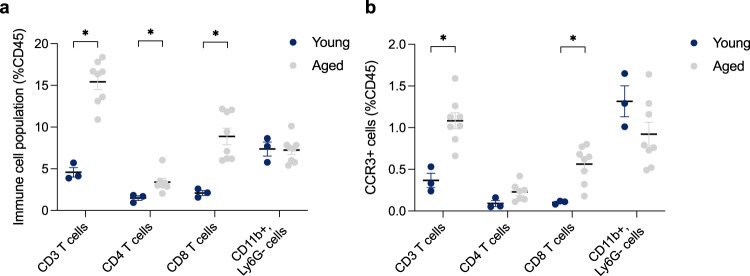
CCR3-expressing T cells increase with age in the choroid plexus. Flow cytometric analysis of surface markers in pooled choroid plexus samples from young (average age of 5 months) and aged (29 month) C57Bl/6 mice. Single cells isolated from pooled choroid plexus samples were stained for markers of interest on T cells (CD3^+^), T helper cells (CD3^+^CD4^+^), Cytotoxic T cells (CD3^+^CD8^+^) and Myeloid cells (CD11b^+^Ly6C^−^). **a** Respective frequencies of cell populations were normalized to total CD45^+^ cells for T cell populations and Myeloid cell populations (*p < 0.05, multiple Mann–Whitney test, *n* = 3, 8). **b** Flow cytometric analysis of CCR3-expressing cells normalized to total CD45^+^ cells in T cell populations and Myeloid cell populations (*p < 0.05, multiple Mann–Whitney test, *n* = 3, 8). All data shown are mean ± standard error of the mean.

In order to investigate whether the infiltrating cells present in the choroid plexus expressed CCR3, we performed flow cytometry on choroid plexus tissue from young and aged mice (Fig. 4). Out of all CD45^+^ cells present, we found only significantly increased T cells in the choroid plexus of aged mice, suggesting increased recruitment and infiltration with age. Of these T cells, 15% were CD3^+^, 4% were CD4^+^, and 10% were CD8^+^. While there was an increase in the relative number of T cells in the choroid plexus of aged mice, the proportion of CCR3-expressing T cells within each subpopulation was unchanged (Supplementary Fig. 5a). However, there was still a significant increase in the numbers of CCR3^+^ T cells in the choroid plexus with age (Fig. 4b). The choroid plexus has more T cells compared to other brain regions (Fig. 3c), however still fewer than those found in blood. When taking into account that ~20% of all circulating T cells express CCR3 (Fig. 2g), 1% of T cells expressing CCR3 in the choroid plexus is a substantial twofold increase in aged mice compared to young mice. After factoring in the threefold increase of T cells (as a percentage of parent CD45^+^ cells) in the choroid plexus, from 5% in young mice to 15% in aged mice, there was in fact a sixfold increase in CCR3-expressing T cell levels in the choroid plexus of aged mice compared to young mice. This is further illustrated by raw cell counts, without normalization, from the choroid plexus (Supplementary Fig. 6). Surprisingly, there was no increase in the CD11b^+^ myeloid lineage, representative of monocytes, eosinophils, and macrophages, in the choroid plexus with age and no change in the proportion of CCR3^+^ cells within this population (Fig. 4a, b, Supplementary Fig. 5b, Supplementary Fig. 6). This suggests that T cells are most likely the primary source of immune cells infiltrating into the brain with age, and a significant proportion of these cells are shown to express CCR3.

Since our single cell RNA-seq transcriptomic data on mouse brains showed *Ccr3* expression in microglia (Fig. 2d), we next asked whether CCR3 inhibition could affect neuroinflammation in the brain due to infiltrating macrophages. Iba1^+^ staining, which identifies both resident microglia and infiltrating macrophages, trended toward a decrease with AKST4290 treatment in aged mice; however, there was no difference observed in TMEM119^+^, a resident microglia-specific marker, suggesting that there may be a minor role for CCR3 inhibition on infiltrating macrophages specifically (Supplementary Fig. 7a, b). There was a significant correlation between CD3^+^ T cells and Iba1^+^ cells in aged mice, while there was no correlation between CD3^+^ and TMEM119^+^ cells, implicating a close relationship between infiltrating T cells and infiltrating macrophages but not with resident microglia (Supplementary Fig. 7c, d). The profound effects on infiltrating T cells into the brain indicates that CCR3^+^ T cells may play a critical role in the process.

### CCR3 inhibition reduces T-cell infiltration in a MOG model

**Fig. 5:**
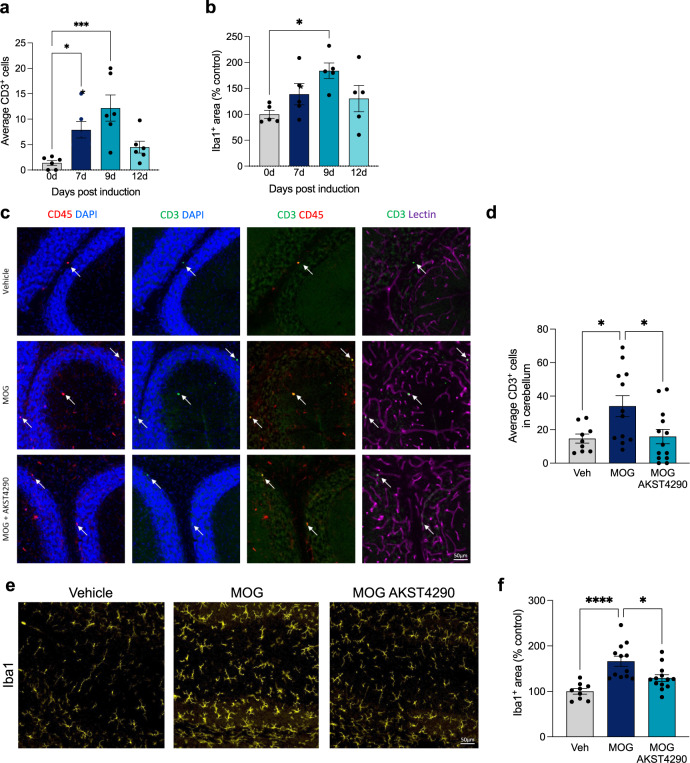
CCR3 inhibition reduces infiltration of T cells into the brain in a model of induced autoimmune inflammatory disease. **a** Quantification of average CD3^+^ T cells in the cerebellum of 2-month-old mice at 0, 7, 9, and 12-days following myelin oligodendrocyte glycoprotein 35–55 (MOG) induction (*n* = 6). Data shown as average CD3^+^ T cell count across 4 sagittal cerebellum sections for each mouse (*p < 0.05, one-way ANOVA). **b** Relative Iba1^+^ signal in the cerebellum of 2-month-old mice at 0, 7, 9, and 12-days following MOG induction (*n* = 5). Data shown as percent area of the region of interest (*p < 0.05, one-way ANOVA). **c** Representative images of CD3^+^ T cells co-stained with CD45 and vessel marker Lectin in the cerebellum. **d** Average number of CD3^+^ T cells in the cerebellum (*p < 0.05, one-way ANOVA; *n* = 9, 12, 13). **e** Representative images of Iba1^+^ microglia in the cerebellum. **f** Relative Iba1^+^ signal in MOG-injected mice with AKST4290 treatment (*p < 0.05, ****p < 0.0001; one-way ANOVA; *n* = 9, 12,13). All data shown are mean ± standard error of the mean.

Due to the mild chronic nature of inflammation in the aged mouse model, or inflammaging, which may not allow direct observation of the catalytic role of CCR3 expressing immune cells, we tested the impact of CCR3 inhibition in a more aggressive inducible model of T-cell infiltration specifically. We immunized young male and female mice with myelin oligodendrocyte glycoprotein 35–55 (MOG) in Incomplete Freund’s adjuvant to induce T-cell infiltration (Fig. 5). We identified an acute timepoint where we could observe a peak in both infiltrating T cells and Iba1^+^ microgliosis, prior to onset of any clinical motor symptoms associated with this model, in order to utilize the model strictly for T-cell infiltration but without the added complexity of clinical EAE symptoms (Fig. 5a, b). We found that AKST4290 treatment reduced T-cell infiltration in the brains of MOG-induced mice, as well as the increased Iba1^+^ signal in these mice (Fig. 5c–f). Thus, in this acute, much more aggressive model, CCR3 inhibition with AKST4290 treatment was able to significantly reduce the infiltration of both T cells and macrophages. In addition, we also found a significant increase in T-cell infiltration in the choroid plexus of young mice treated with recombinant CCL11, which was reduced by ~50% with AKST4290 treatment although not statistically significant (Supplementary Fig. 8). The indirect effects of CCR3 inhibition, in the absence of CCL11, on the MOG-induced T cells mirror the direct effects observed with recombinant CCL11-induced T cells. Taken together, these data demonstrate the robust effect of CCR3 inhibition on peripheral T-cell infiltration into the brain, with three distinct but complementary models, and present a CCR3-dependent mechanism for recruitment of immune cells into the brain in aging and disease.

## Discussion

T-cell infiltration from the periphery to the brain has long been thought to be an important component of a wide range of neurodegenerative diseases with known blood-brain barrier dysfunction, such as multiple sclerosis, amyotrophic lateral sclerosis, and Parkinson’s disease^35–42^. The recruitment of T cells into the brain can be initiated by disease-specific pathology such as alpha-synuclein, by chemotaxis due to increased cytokine expression in the brain, or by general leakiness of the blood-brain barrier in disease. Recent studies have demonstrated the existence and importance of T-cell infiltration in the absence of excessive penetrance or leakage of the blood-brain barrier, such as in Alzheimer’s disease and even in normal aging^23,43^, suggesting that there is more crosstalk between the brain and the periphery than originally thought. This event may be the catalyst for subsequent inflammation, offering one hypothesis for the vulnerability of the aged brain to a broad spectrum of diseases.

The chemoattractant role for CCL11 in CCR3-mediated T-cell migration has been described previously in peripheral tissues^19,32–34^, but less understood is the role of CCR3 and T cells in diseases of the CNS. Here we clearly demonstrate that there is upregulation of a wide spectrum of chemokines with aging and targeting their receptors is expected to provide an effective therapeutic target with CNS benefits. Many of these chemokines are already reported to be linked to aging or disease. CCL2, for example, is associated with a faster cognitive decline in prodromal Alzheimer’s disease patients^43^, and here we show that CCL11 and CCL24 are also critical inflammatory mediators of cognitive dysfunction. Targeting CCR3, the central receptor of these chemokines, presents a unique and attractive strategy for improvement of chemokine-induced CNS deficits. In support of our findings, peripheral CCR3-mediated T-cell chemotaxis has been demonstrated both directly (through CCR3-expressing T cells) and in conjunction with CCR3-expressing eosinophils such as in tumorigenesis^44^. Interestingly, in models of skin, lung, or gastrointestinal inflammation, CCR3^+^ T lymphocytes are recruited together with eosinophils, again suggesting an interaction between these cell types in disease^44^. Other studies have shown the ability CCL11 to recruit T cells to tissue sites further supporting a role for CCR3 and CCL11 in T cell migration^5^. However, it is unknown whether these same mechanisms take place in the brain and future studies are warranted to uncover the precise nature of these cell-type specific effects. Furthermore, the direct effect of infiltrating T cells on neuronal function is yet to be determined even though multiple studies have indeed demonstrated a link between T cells and cognitive function^45,46^, highlighting the impact that the crosstalk between immune cells can have on behavior and function. Taken together, our data show that CCR3 is a profound contributor to T-cell infiltration and microgliosis in the brain, which can have profound consequences on cognitive and motor function. Inhibition of CCR3 is expected to provide a robust therapeutic approach for the treatment of aging-associated neuroinflammatory diseases, particularly those associated with excessive T-cell infiltration such as multiple sclerosis and Parkinson’s disease.

## Methods

### Animals

C57Bl/6 mice were acquired from Jackson Laboratories. Experiments were performed on young mice at 2–3 months old and on aged mice at 20–24 months old. All mice used were male, with the exception of MOG-induced T cell model, which included males and females. All mice were housed in plastic cages on a 12 h light cycle with ad libitum access to water and a standard laboratory diet unless otherwise noted. All studies were performed in accordance with the Alkahest Institutional Animal Care and Use Committee using the National Institute of Health guidelines.

### Materials and reagents

AKST4290 was synthesized at Boehringer Ingelheim and formulated in 33.75% 2-hydroxypropyl-β-cyclodextrin and adjusted to pH 6.5 with 1 M sodium hydroxide (NaOH) at 15 mg/mL and dosed at 30 mg/kg for experiments with p.o. (oral gavage) administration. Compound and vehicle solutions were prepared weekly and stored at 4 °C.

For all chronic studies with more than 3 weeks of treatment, AKST4290 was administered via drinking water. Compound was formulated weekly at 1 mg/mL in water (M-WB-300A, Innovive) containing 2% sucrose and 0.1% potassium sorbate, and pH was adjusted to 6.5 with 1 M NaOH before use. Drinking water bottles were protected from light throughout the administration period.

Recombinant mouse proteins CCL11 and CCL24 were dissolved in phosphate-buffered saline (PBS) and administered at 10 µg/kg and 40 µg/kg, respectively. A working solution of 0.7 µg/mL was prepared from a stock solution of 1 µg/µL. Solutions were made fresh weekly and stored at 4 °C.

### Behavior assays

#### Rotarod

Mice were placed on the rods and allowed to sit for 30 s. Then the rods were rotated at a constant speed of 5 rpm for 5 min. If a mouse fell off, it was placed back on until it completed 5 min without falling off. If a mouse was unable to complete the training, it was excluded from testing. For testing, mice were placed on the rods and allowed to sit for 30 s. Then the rods were rotated at a ramp of 90 s from 5 rpm to 40 rpm. The time at which they fell off was recorded. This was repeated two more times for a total of 3 trials. The minimum inter-trial interval was 15 min.

#### Barnes maze

A modified Barnes Maze was used to assess spatial working/episodic learning and memory in mice. The Barnes Maze apparatus is a circular maze ~95 cm off the ground with a diameter of 118 cm. There are 40 escape holes on the surface of the maze, each with a diameter of 5 cm placed along three rings of varying distances from the center of the platform. An escape box is attached to one of the holes and all holes are left uncovered. Bright lights and a fan are trained on the maze to provide aversive stimuli and encouragement to find the escape hole. Visual cues are placed on all 4 sides of the maze. All mice were given 4 trials a day to find the escape hole, with ~10 min between each trial. For young mice the maximum duration for a trial was 90 s on each day, whereas for aged mice the maximum duration was 90 s on the first day and 120 s for the following 3 days. Mice that could not complete the task in that time were guided to the escape hole where they could enter and stay for 30 s before being returned to their home cage. Mice were divided into groups of 4–5 mice each, with balanced treatment groups. Group 1 mice were run for 4 trials, then Group 2 mice were run for 4 trials, and so on until all groups finished testing. Mouse activity was tracked using CleverSys analyzing software and the events recorded included velocity, distance moved and escape latency. At the end of each trial, the maze and escape hole were wiped with 70% ethanol.

#### Radial arm water maze

The water maze was filled with water at least 24 h prior to the test to equilibrate to 24 °C. The water was dyed with white latex paint to make the animals visible for tracking and to allow for the use of a hidden platform. Eight distinct visual cues were placed at the end of each arm of the RAWM inserts. On day 1, young mice and aged mice were given 6 and 5 trials respectively, with a visible platform and a 30–60 min inter-trial interval. Animals had 60 s to reach the platform. If they did not reach the platform in that time, they were guided to it and allowed to remain for 15 s before being removed from the tank. The goal arm remained constant and a different start arm was randomly assigned for each of the 5 trials so that mouse started in every arm once except for the 2 arms directly across from the platform. The goal arm was switched after every 2 mice and balanced between all treatment groups. After each trial, the mice were placed in an empty cage with blue pads and allowed to dry off under a heat lamp before being placed back into their home cage. Day 2 was the testing day conducted 48 h after training, where animals were subjected to the same test of 5 or 6 trials each and a 30–60 min inter-trial interval, but with a hidden platform. Animals were scored for number of errors (entry into a non-goal arm) and for latency to reach the platform. All trials were recorded using either Top Scan or ANY-maze.

#### Contextual fear conditioning

Mice were brought into the testing room immediately before their trial to avoid exposure to sounds and scents from testing. Day 1: For training, mice were placed in the chambers, bright house light and fan on, for 2 min. Then an auditory cue (2000 Hz, 70 dB, conditioned stimulus [CS]) was presented for 30 s. A 2 s foot shock (0.6 mA; unconditioned stimulus [US]) was administered for the final 2 s of the CS. This procedure was repeated once, each after a 2 min interval, and the mouse was removed from the chamber 30 s after the second shock. The pans, chamber walls, and grid floors were cleaned with 70% ethanol between trials. Day 2: 72 h after the training, the mouse was returned to the same chamber in which the training occurred (memory for context), and freezing behavior was recorded for 3 min. The mouse was returned to its home cage. The pans, chamber walls, and grid floors were cleaned with 70% ethanol between trials.

### Single cell RNA-seq

Single cell suspensions were obtained by micro-dissecting Dentate gyri from brains of both young (2 months) and aged (22 months) mice using Miltenyi Neural Tissue Dissociation kit (postnatal neurons; Miltenyi Biotec). Live cells were fluorescence activated cell sorted (unbiased) using a SONY MA900 and a SONY SH-800 sorter (Sony Biotechnologies, San Jose, CA) and collected into 96 well plates for single cell RNA-seq using SMART-Seq v4 (Takara, Mountain View, CA) following the manufacturer’s protocol, except half-reaction volumes were used. Libraries (Nextera XT; Illumina, San Diego, CA) were generated following manufacturer’s protocol and sequenced on either a HiSeq 4000 or NovaSeq following standard protocols. Sequencing reads were processed and aligned against Gencode M16 using standard tools including cutadapt, STAR, and RSEM. Downstream processing, cell classification, and differential expression analysis between aged and young cell types was performed using Seurat^47^. Differential gene expression analysis was carried out with the Seurat package using the MAST package^48^. Finally, raw *p* values were Benjamini–Hochberg corrected^49^.

### Immunohistochemistry

Mice were anesthetized with Avertin (2,2,2-tribromoethanol, T48402-25G, Sigma Aldrich) diluted in sterile saline at a final concentration of 0.04 g/mL and given at a concentration of 125–250 mg/kg. Mice were perfused with 1% EDTA in PBS transcardially, and brains were dissected and hemibrains were fixed in 4% paraformaldehyde (PFA) solution for use in immunohistochemistry. After 2 days of fixation, the hemibrains were transferred to a 30% sucrose in PBS solution and changed after 2 days. Hemibrains were sectioned coronally at 30 µm on a microtome at −22 °C. For the MOG study, mice were perfused with 4% PFA solution immediately following PBS and hemibrains were sectioned sagittally at 35 µm on a microtome. Brain sections were stored in cryoprotectant media containing 30% ethylene glycol (E178-4, Fisher Scientific), 30% glycerol (G5516, Sigma Aldrich), 11.4 mM sodium phosphate monobasic monohydrate (S9638-500G, Fisher Scientific) and 38.5 mM sodium phosphate dibasic heptahydrate (S9390-500G, Fisher Scientific) in MilliQ water at −20 °C until needed for staining. All brain sections were processed for immunohistochemistry as free-floating sections in the appropriate serum at 10% in phosphate-buffered saline with Tween® 0.5%. Primary antibodies were incubated overnight at 4 °C or room temperature as described below.

Rat anti-CD3 (555273, BD Biosciences) was used at a concentration of 1:100 at room temperature, rabbit anti-CD45 (702575, Cell Signaling) was used at a concentration of 1:200 at room temperature, rabbit anti-Iba1 (016–26721, Wako) was used at a concentration of 1:1000 at 4 °C, guinea-pig anti-TMEM119 (400 004, Synaptic Systems) was used at a concentration of 1:200 at 4 °C, and Dylight 488/594/697 labeled Lycopersicon Esculentum (Tomato) Lectin (DL-1177, Fisher Scientific) was used at a concentration of 1:200 at room temperature. The appropriate fluorescent secondary antibodies (Alexa-488/555/647, Invitrogen) were applied the next day at a concentration of 1:300 for 1 h at room temperature. Prolong Gold Mounting Media was used to coverslip the slides. Images were acquired on the Hamamatsu Nanozoomer 2.0HT slide-scanner at 20x.

CD3 and CD45 positive cells were counted from images acquired on the Hamamatsu slide scanner. The total number of positive cells was averaged from up to 3, 5, and 8 sections for each mouse from choroid plexus, SVZ, and hippocampus, respectively. Ordinary one-way analysis of variance (ANOVA) was used to test for statistical significance with Dunnett’s multiple comparisons test post-hoc between 3 treatment groups, while a *t-*test was used to test for significance between 2 treatment groups.

Iba1and TMEM119 positive areas were quantified using a fixed threshold on Image Pro Premier v9.2 software as a percentage of an ROI around the entirety of specific brain regions. The positive percent area was averaged from approximately five sections for each mouse. Ordinary one-way ANOVA was used to test for statistical significance with Dunnett’s multiple comparisons test post-hoc between 3 treatment groups, while a *t*-test was used to test for significance between 2 treatment groups.

### Flow cytometry

Blood was collected via cardiac puncture into an EDTA-coated syringe and 100 µL aliquoted into flow cytometry tube at room temperature. All samples were processed for flow cytometry at the same time. Red blood cells were lysed with 1X lyse/fix buffer (558049, BD Biosciences) for 10 min at room temperature (RT) followed by a spin at 500 g for 8 min RT. The supernatant was discarded, cells were washed in Hanks’ balanced salt solution (HBSS) followed by another spin at 500 g for 8 min RT. The cell pellet was resuspended in 100 μl of stain buffer (554656, BD Biosciences) and incubated at RT with 1 µL of the following antibodies for 30 min in the dark; anti-CD45-FITC (103108, BioLegend), anti-CD170-AF 700 (56-1702-82, ThermoFisher), and 2 µL of the following antibodies; anti-CD3-PerCP-efluor710 (46-0032-82, ThermoFisher), anti-CCR3 AF647 (FAB1551R, Novus), anti-CD4 eFluor 506 (69-0042-82, ThermoFisher), anti-CD8 Super Bright 600 (63-0081-82, ThermoFisher), anti-Ly-6G (Gr-1) PE-eFluor 610 (61-9668-82, ThermoFisher), and anti-B220 (CD45RT) PE-Cyanine7 (25-0452-82, ThermoFisher). An extra sample was processed including all antibodies except anti-CCR3, as a Fluorescence Minus One (FMO) control. Cells were then washed in stain buffer and the final pellet resuspended in 300 μl of stain buffer. Data were acquired on an Attune NxT Flow Cytometer (ThermoFisher) and analyzed with FlowJo Analysis Software (Treestar, v10.8) with the gating strategy described in Supplementary Fig. 4.

### Isolation and dissociation of choroid plexus for flow cytometry

Choroid plexuses were isolated from the lateral ventricles and fourth ventricles of 2 mice and pooled into an Eppendorf tube containing 400 µL of ice cold HBSS. Tissues were minced with micro scissors and dissociated in the presence of DNaseI (Sigma-Aldrich, 30 U/mL), Collagenase I (Worthington Biochemical, 10 U/mL), and Collagenase IV (Worthington Biochemical, 400 U/mL) for 30 min in a 37 °C water bath. Every 10 min, tubes were agitated with a pipet to ensure proper dissociation. Samples were resuspended with HBSS, passed through a pre-wet 70 µm mini-strainer flow cytometry tube (PluriSelect Life Science, Leipzig, Germany) and washed with HBSS. Flow tubes were spun down at 500 g for 5 min at 4 °C, aspirated, and resuspended in antibody stain for 30 min on ice in the dark. The antibody stain consisted of 88 µL of BD Brilliant stain buffer (563794, BD Bioscience) and 1 µL of each of the following antibodies; anti-CD45-FITC (103108, BioLegend), anti-CD11c-APC (20-0114-U100, Tonbo Bioscience), anti-CD3-PerCP-efluor710 (46-0032-82, ThermoFisher), anti-CD8a-APC/Cyanine7 (100714, Biolegend), anti-I-A/I-E Brilliant Violet 421 (107632, Biolegend), anti-CD11b Brilliant Violet 711 (101242. Biolegend), anti-CCR3 PE (FAB1551P, Novus Biologicals), anti-CD4 PE/Cyanine7 (100422, Biolegend), anti-CD19 Brilliant Violet 605 (115540, Biolegend), and anti-Ly6G Brilliant Violet 605 (127639, Biolegend). A blood sample from one animal was collected and processed with the same antibody panel to use as a staining control. Cells were then washed and the final pellet resuspended in 400 μl of stain buffer. Data were acquired on an Attune NxT Flow Cytometer (ThermoFisher) and analyzed with FlowJo Analysis Software with the gating strategy described in Supplementary Fig. 9.

### MOG-induced T-cell infiltration model

MOG 35-55 (Anaspec AS-60130-5) was reconstituted in PBS at 4 mg/mL. M. Tuberculosis H37 Ra (Fisher Scientific DF3114-33-8) was dissolved in Incomplete Freud’s adjuvant (Sigma-Aldrich, F5506) at 4 mg/mL. The two solutions were then emulsified using glass syringes (Thermo Scientific Male Luer-LOK Priming Syringes 03-170-301) and 3-way stop cock (Cadence 6001). Solutions were prepared just before dosing. Mice were dosed with 50 µL of the emulsion SQ on each flank (100 µL total) using a glass syringe and 21 G needle. Pertussis toxin (Sigma P7208-50UG) was dissolved at 0.002 mg/mL in saline and injected 100 µL intravenously on the first day of induction and again 2 days later. Mice were treated with either vehicle or AKST4290 (30 mg/kg) p.o. twice per day for 9 days starting on the day of induction with MOG.

### Measurement of AKST4290

AKST4290 drug levels were measured from plasma, CSF, or brain samples by liquid chromatography-tandem mass spectrometry (LC-MS/MS) at Quintara Biosciences (Hayward, CA). For measurement from CSF, CSF was collected from the cisterna magna following deep anesthetization and snap frozen. For measurement from brain, cortex was sub-dissected and snap frozen. Cortex samples were homogenized with 3 volumes of ice-cold water. An aliquot of 5 µL of plasma, CSF, or cortex homogenate was added to 5 µL of 70% acetonitrile followed by extraction with 100 µL of acetonitrile containing internal standard (50 ng/mL of Dexamethasone). The mixture was vortexed on shaker for 15 min and subsequently centrifuged at 4000 *rpm* for 15 min. An aliquot of 50 µL of the supernatant was mixed with 70 µL of 0.1% formic acid in water for the injection to the LC-MS/MS.

### MARG (Microautoradiography)

Microautoradiography experiments were performed at Invicro (Boston, USA). Mice aged 2 months and 20 months were dosed with either vehicle or ^14^C-AKST4290 30 min prior to euthanasia to determine brain penetrance of AKST4290. Brains were frozen and embedded in optimal cutting temperature (OCT) medium and sectioned at 10 μm thickness to assess presence of ^14^C-AKST4290 signal. Collected sections were mounted directly on emulsion-coated glass slides in the dark and remained in a lead covered box at −20 °C until sections were fixed upon development. Slides were developed with Kodak D19 developer solution and Kodak Kodafix Fixative solution and counterstained with freshly prepared and filtered methylene blue basic fuchsin (MBBF) before cover-slipping with SubX Permount (Leica) mounting media. All slides were imaged under Pannoramic DESK single slide scanner and data was viewed using CaseViewer to qualitatively analyze and report images.

### Autoradiography

Autoradiography (ARG) experiments were performed at Invicro (Boston, USA). ARG data was analyzed using CiQuant software (Invicro). ARG plates were calibrated using the known standard concentrations. Individual ARG sections were cropped from their respective plate images and decay corrected back to the time of injection. All ARG sections and white light (WL) images were then co-registered via rigid and affine transformations respectively. Images from ARG were analyzed for tracer concentration in brain and eye sections. To this purpose, brain regions of interest (ROIs) were defined manually based on both the WL image and the ARG signal when present. For eyes, ROIs were defined around lens, vitreous humor, and uveal tract.

### Proteomic analysis of CCR3 ligands

Plasma samples were collected from healthy male donors of 18, 30, 45, 55, and 66 years of age by plasmapheresis as part of standard plasma collection for Grifols Biomat USA source plasma. “Retain tubes” of individual donations containing ~3 mL plasma were stored at −80 °Cat Grifols and shipped frozen to Alkahest. Only samples from “infrequent donations” (collected >60 days after preceding plasma donation) were used.

Plasma samples of 40 individuals per age group were thawed on ice-water and pooled into 8 pools of 5 samples each. Resulting pools were centrifuged at 3200 × *g* for 30 min at 0 °C and filtered through 0.22 um Millex GV filter (MilliporeSigma, Burlington, MA) to remove cryoprecipitate. Filtrate was aliquoted into cryotubes and stored at −80 °C until use.

Relative concentrations of 981 unique proteins (all analytes of 12 Olink panels) were measured in the plasma samples by Proximity Extension Assay technology (PEA) (Assarsson) at Olink Analysis Service (Watertown, MA). The PEA immunoassay relies on simultaneous dual target recognition by matched antibody pairs that are labelled by complementary oligonucleotide sequences. When both antibodies bind to the target protein, the oligonucleotide tags hybridize, and extension results in a DNA reporter which is quantified by real‐time qPCR. Protein levels are reported in NPX (Normalized Protein eXpression), Olink’s arbitrary unit which is in log2 scale. Assay characteristics and validation data (e.g., detection limit, precision, validated sample types) are available from the Olink website (https://www.olink.com/).

### Statistics and reproducibility

All data were assessed for normal distribution by Shapiro–Wilk test and appropriate parametric or non-parametric test was used to test for statical significance. Statistical tests were performed using Prism for all data except single cell RNA-seq data, for which differential gene expression analysis was carried out with the Seurat package using the MAST package and raw *p* values were Benjamini–Hochberg corrected, as described in the section above. Comparisons between two groups were assessed by an unpaired *t*-test or Mann–Whitney test, for parametric and non-parametric data respectively. Comparisons between three groups or more were assessed by one-way ANOVA and Kruskal–Wallis test. Comparisons between groups in behavioral tests with repeated measures, like the Barnes Maze or RAWM, were assessed by Marginal Survival Analysis and Cox Proportional Hazards. Statistical analyses utilized individual animals as replicates and these are presented as datapoints on all graphs except for flow cytometry data from choroid plexus, which utilized pooled samples. Behavioral tests utilized a sample size of 10 or more per group and histological or flow cytometry analyses utilized sample size of 5 or more per group. Statistical significance was defined as a *p* value of <0.05.

### Reporting summary

Further information on research design is available in the Nature Portfolio Reporting Summary linked to this article.

## Supplementary information

Supplementary Information

Description of Additional Supplementary Files

Supplementary Data

Reporting Summary

## Data Availability

The data that support the main findings of this study are available in the Supplementary Data file. Data for single-cell RNA-seq is available through data repository (Fig. 1b: 10.6084/m9.figshare.22137980, Fig. 2d: 10.6084/m9.figshare.22138001) Data for findings in the Supplementary Information are available from the corresponding author upon reasonable request.
