# Supplementary material for: CCR3 plays a role in murine age-related cognitive changes and T-cell infiltration into the brain
Source: Commun Biol. 2023 Mar 18;6:292. doi: 10.1038/s42003-023-04665-w (PMC10024715; doi:10.1038/s42003-023-04665-w)
Supplement: Supplementary file 1 — Supplementary Information [file 42003_2023_4665_MOESM1_ESM.pdf]

SUPPLEMENTARY FIGURES

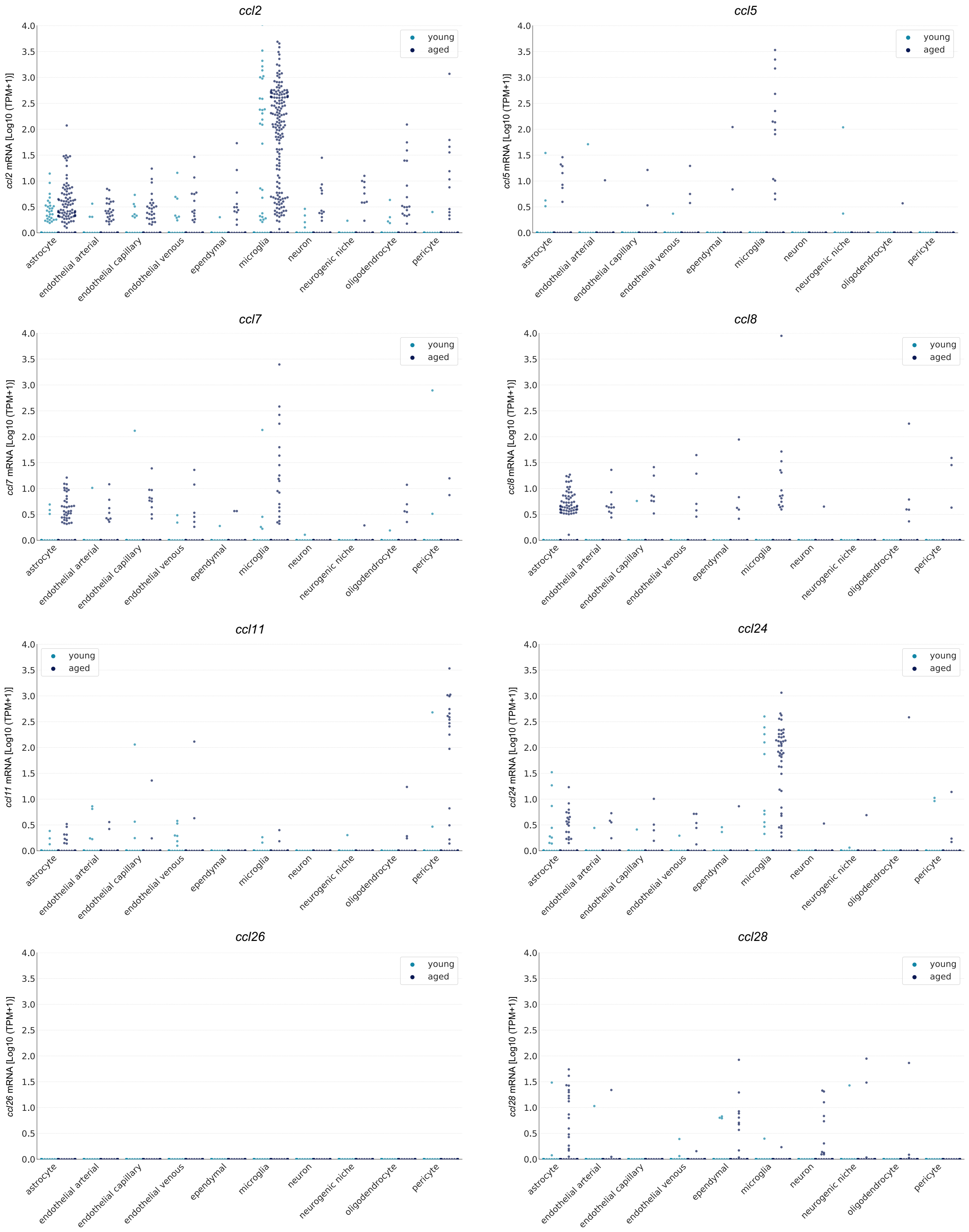

Supplementary Figure 1. Single cell RNA-seq expression of CCR3 ligands.

Panels show expression values in transcripts per million (TPM) for major cell types characterized. Each panel shows the expression distribution for one of the chemokines *ccl2, ccl5, ccl7, ccl8, ccl11, ccl24, ccl26* and *ccl28*. For each gene, expression levels are grouped by cell type and by age. Cells harvested from young dentate gyri are shown in light blue, cells from aged dentate gyri in dark blue.

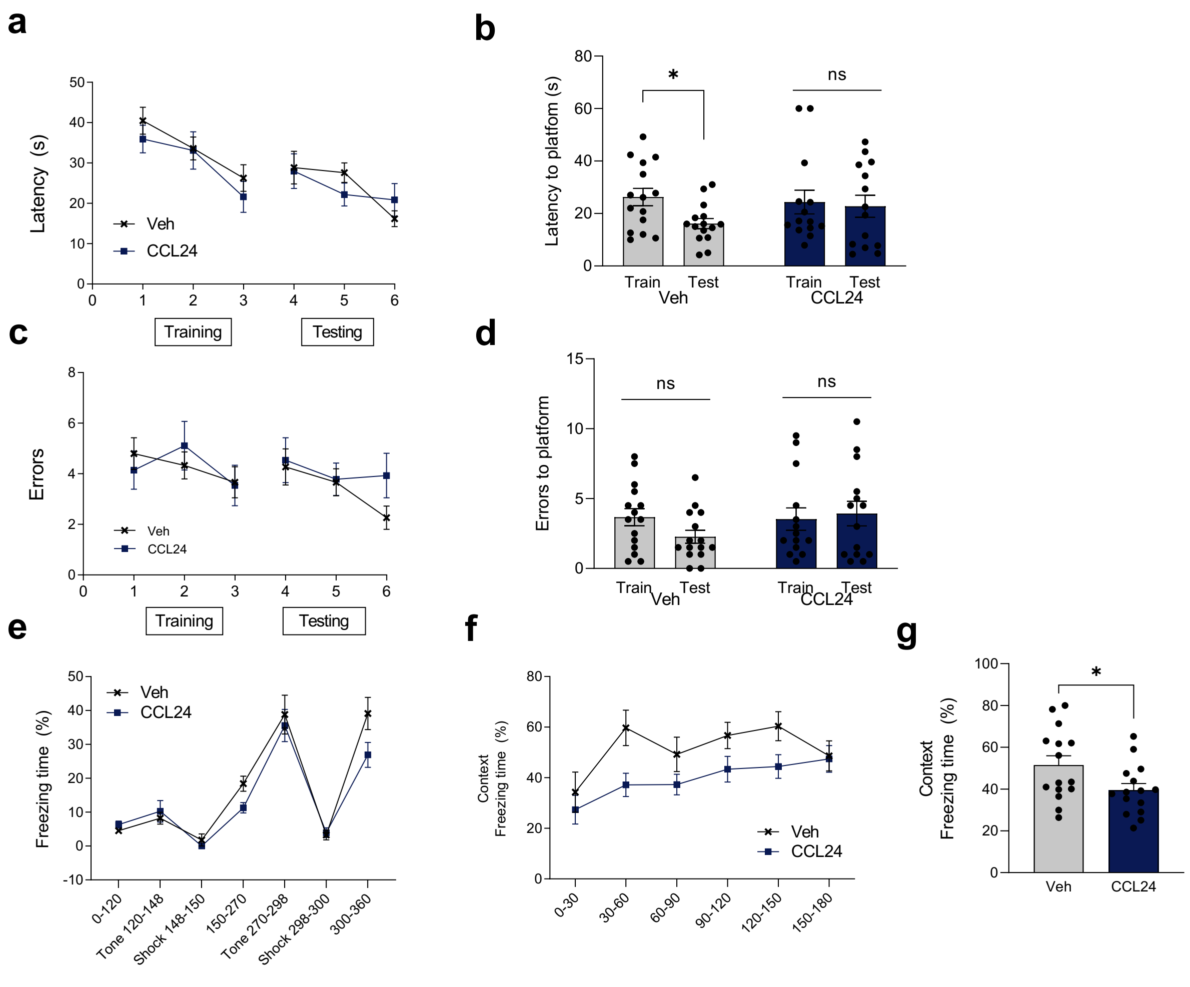

Supplementary Figure 2. CCL24 induces cognitive deficits in young mice.

(**a**) Average latency across all training and testing trials in RAWM test (n=15). (**b**) Average latency of last two training and testing trials in RAWM test (*p<0.05, paired t-test; n=15). (**c**) Average number of errors across all training and testing trials in RAWM test (n=15). (**d**) Average errors of last two training and testing

trials (n=15). (**e**) Average percent time freezing time in training bins (s, seconds) across training in the fear conditioning test (mixed effects analysis; n=15, 14). (**f**) Average percent time freezing in 30 second (s) bins across context testing (mixed effects analysis; n=15, 14). (**g**) Total percent time freezing averaged across the entire 180 second contextual test (**p*<0.05, unpaired t-test; n=15, 14). All data shown are mean ± standard error of the mean.

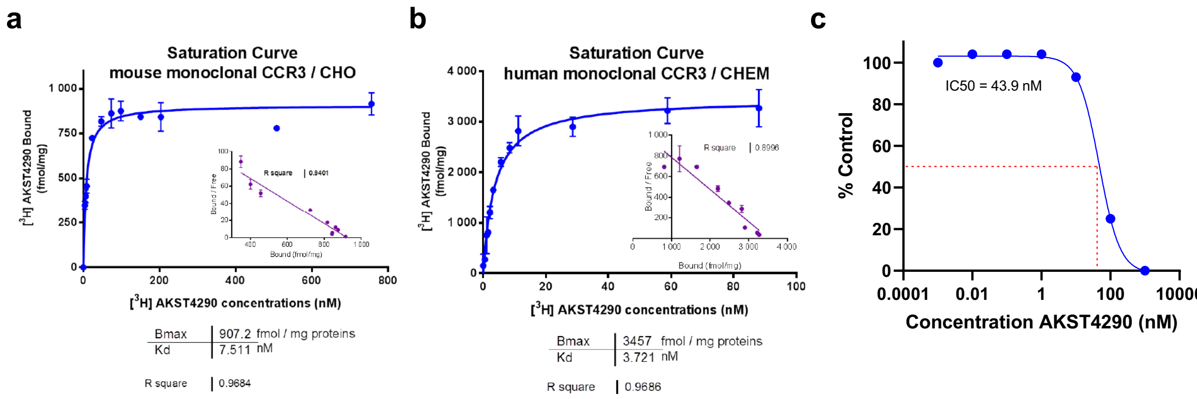

Supplementary Figure 3. On and off-target pharmacology of AKST4290.

In vitro dose response in mouse (**a**) and human (**b**) – direct binding of [3H]-AKST4290 to mouse CCR3 receptor. (**c**) In vitro dose response in L1.2 cells overexpressing mouse CCR3 in a chemotaxis assay in response to 1 nM mouse eotaxin.

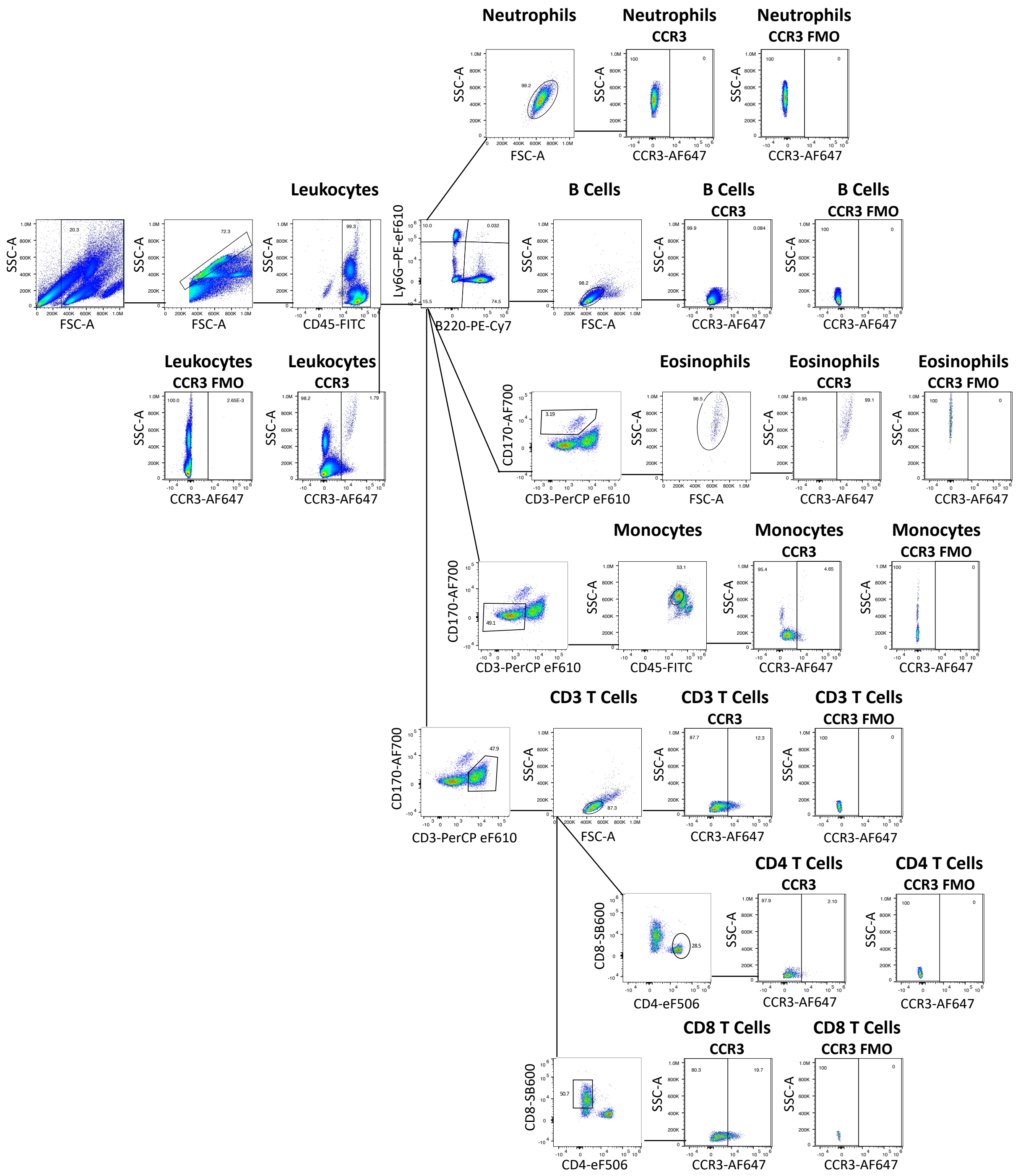

Supplementary Figure 4. Flow cytometry gating strategy for whole blood.

A gating strategy was used to identify immune cell populations and CCR3 expression via flow cytometry.

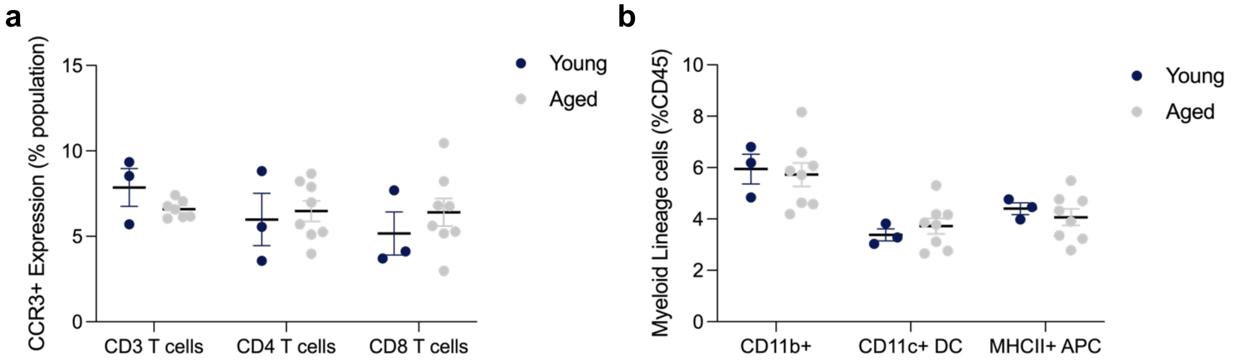

Supplementary Figure 5. CCR3 expressing T cell subpopulations and myeloid lineage cells in choroid plexus.

Flow cytometric analysis of surface markers in pooled choroid plexus samples from young (average age of 5 months) and aged (29 month) C57Bl/6 mice. Single cells isolated from pooled choroid plexus samples were stained for markers of interest on T cells ($CD3^+$), T helper cells ($CD3^+CD4^+$), and Cytotoxic T cells ($CD3^+CD8^+$), Myeloid cells ($CD11b^+$), Dendritic cells ($CD11b^+CD11c^+$), Antigen Presenting Cells ($CD11b^+MHCII^+$). (a) Respective frequencies of CCR3 expressing cells were normalized to the parent T cell populations. (b) Respective frequencies of cell populations were normalized to total $CD45^+$ cells for Myeloid lineage cell populations. (d). All data shown are mean ± standard error of the mean.

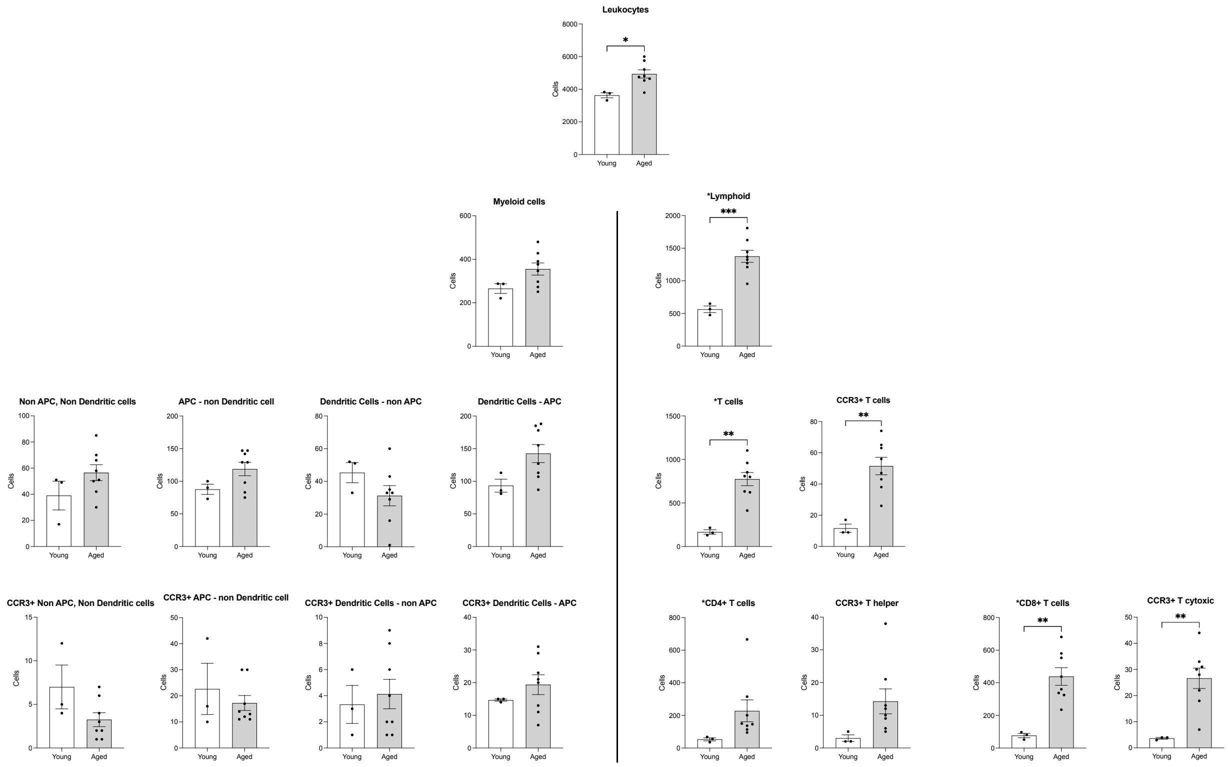

Supplementary Figure 6. Cell counts of CCR3-expressing T cell subpopulations and myeloid lineage cells in choroid plexus.

Flow cytometric analysis of surface markers in pooled choroid plexus samples from young (average age of 5 months) and aged (29 month) C57Bl/6 mice. Single cells isolated from pooled choroid plexus samples were stained for markers of interest on T cells ($CD3^{+}$), T helper cells ($CD3^{+}CD4^{+}$), Cytotoxic T cells ($CD3^{+}CD8^{+}$), Myeloid cells ($CD11b^{+}Ly6C^{-}$) and various Myeloid cell subtypes; Dendritic cells (CD11c+, MHCII-), Antigen Presenting Cells (CD11c-, MHCII+). Populations of interest were manually gated in FlowJo software and raw values are shown. Statistical analysis was completed using Prism software (*p<0.05, multiple Mann-Whitney test, n=3, 8). All data shown are mean ± standard error of the mean.

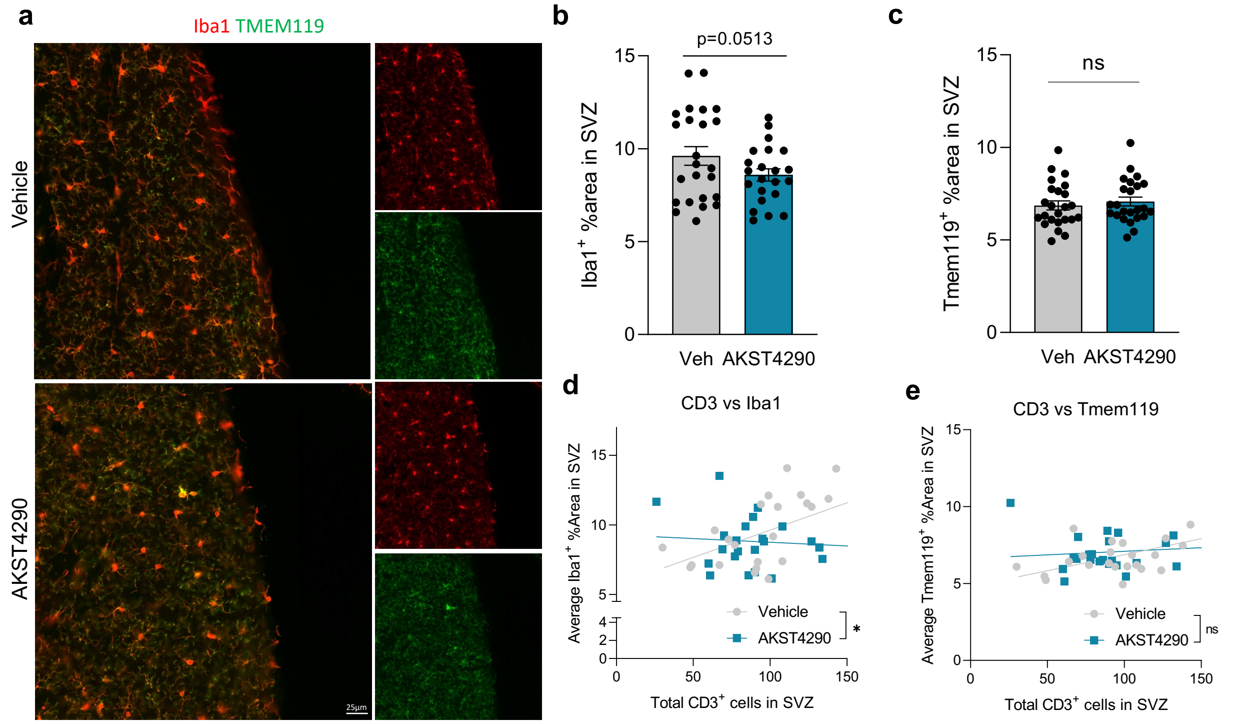

Supplementary Figure 7. CCR3 inhibition effects on microglia and monocytes.

24-month-old C57Bl/6 mice were treated with AKST4290 for 5 weeks. (**a**) Representative images illustrating the staining of resident macrophages (TMEM119$^+$), and all macrophages (Iba1$^+$) in the subventricular zone (SVZ). (**b**) Iba1$^+$ total microglial percent area in the SVZ (unpaired t-test; n=24, 22). (**c**) TMEM119$^+$ resident microglial percent area in the SVZ (unpaired t-test; n=24). (**d**) Correlation plot between Iba1$^+$ microglial percent area and total CD3$^+$ T cells in the SVZ (*****$p<0.00001$, simple linear regression; n=24, 23). (**e**) Correlation plot between TMEM119$^+$ resident microglia percent area and total CD3$^+$ T cells (n=24). All data shown are mean ± standard error of the mean.

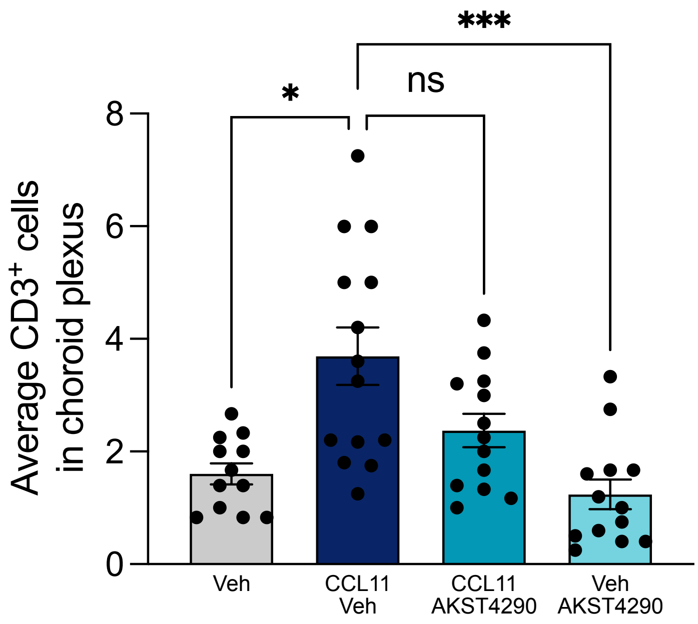

Supplementary Figure 8. Recombinant CCL11 induces T-cell infiltration into brains of young mice.

$CD3^+$ T cell were counted using IHC conducted on 2-month old C57Bl/6 mice treated with vehicle (Veh) (n=11), recombinant CCL11 i.p. (n=15), recombinant CCL11 i.p. with AKST4290 p.o. (n=15), and AKST4290 p.o. (14). Average number of $CD3^+$ T cells in choroid plexus (n=11, 15, 15, 14); *p<0.05, ***p<0.001; one-way ANOVA and Kruskal-Wallis test).

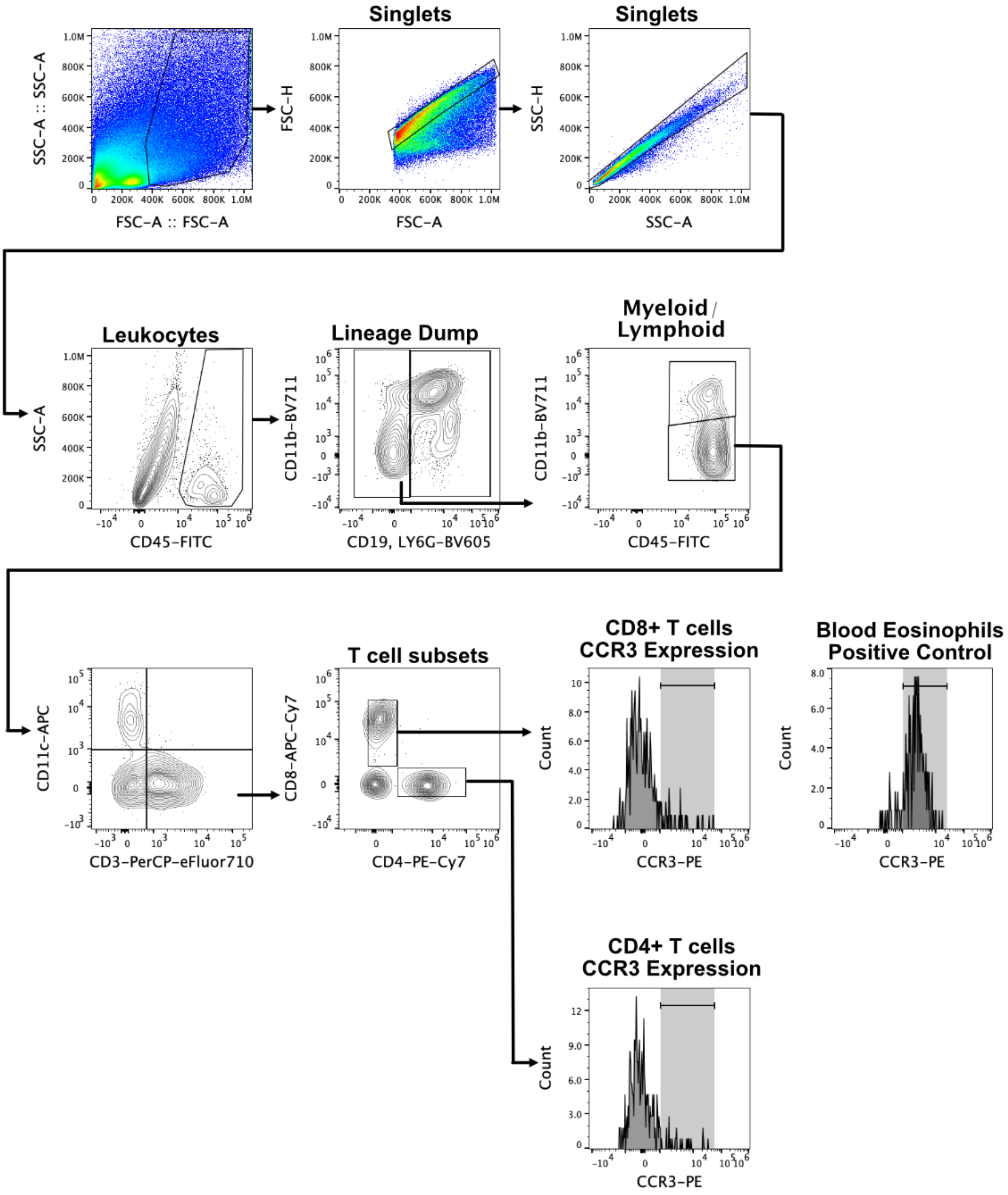

Supplementary Figure 9. Flow cytometry gating strategy for choroid plexus tissue.

A gating strategy was used to identify immune cell populations and CCR3 expression via flow cytometry.

Supplemental Table 1.

Summary of off-target activity of AKST4290 against 80 receptor, ion channel and transporter panel

Assay	Test Concentration (µM)	% Inhibition of Control Specific Binding[1]	IC_{50} (µM)[2]	K_i(µM)[2]
A1 (h)	10	-8		
A2A (h)	10	0		
A3 (h)	10	3		
α1 (non-selective)	10	15		
α2 (non-selective)	10	10		
β1 (h)	10	4		
β2 (h)	10	7		
AT1 (h)	10	-9		
AT2 (h)	10	-9		
BZD (central)	10	6		
BZD (peripheral)	10	-4		
BB (non-selective)	10	1		
B2 (h)	10	-2		
CGRP (h)	10	-3		
CB1 (h)	10	-8		
CCKA (h) (CCK1)	10	-12		
CCKB (h) (CCK2)	10	-22		
D1 (h)	10	10		
D2S (h)	10	14		
D3 (h)	10	2		
D4.4 (h)	10	7		
D5 (h)	10	-17		
ETA (h)	10	-3		
ETB (h)	10	0		
GABA (non-selective)	10	-12		
GAL1 (h)	10	-11		
GAL2 (h)	10	10		
PDGF	10	-6		
TNF-α (h)	10	17		
CCR1 (h)	10	11		
H1 (h)	10	0		
H2 (h)	10	12		
MC4 (h)	10	6		
MT1 (h)	10	22		
M1 (h)	10	13		
M2 (h)	10	15		
M3 (h)	10	0		

M4 (h)	10	25		
M5 (h)	10	5		
NK1 (h)	10	50		
NK2 (h)	10	47		
NK3 (h)	10	10		
Y1 (h)	10	3		
Y2 (h)	10	-2		
NT1 (h) (NTS1)	10	-10		
δ2 (h) (DOP)	10	0		
κ (KOP)	10	27		
μ (h) (MOP) (agonist site)	10	19		
ORL1 (h) (NOP)	10	5		
PACAP (h) (PAC1)	10	-18		
PCP	10	10		
EP4 (h)	10	9		
TP (h) (TXA2/PGH2)	10	-16		
IP (h) (PGI2)	10	-11		
P2X	10	8		
P2Y	10	3		
5-HT1A (h)	10	1		
5-HT1B	10	-1		
5-HT2A (h)	10	-2		
5-HT2B (h) (agonist site)	10	6		
5-HT2C (h)	10	-1		
5-HT3 (h)	10	-1		
5-HT5A (h)	10	3		
5-HT6 (h)	10	-6		
5-HT7 (h)	10	2		
σ (non-selective)	10	16		
sst (non-selective)	10	14		
Glucocorticoid (h) (GR)	10	-5		
VIP1 (h) (VPAC1)	10	-10		
V1a (h)	10	8		
$Ca2^{+}$ channel (L, verapamil site) (phenylalkylamines)	10	-1		
$K^{+}V$ channel	10	-3		
$SK^{+}Ca$ channel	10	-13		
Na^{+} channel (site 2)	10	26		
Cl^{-} channel (GABA-gated)	10	11		
NE transporter (h)	10	-1		
DA transporter (h)	10	42	12	6.5
5-HT transporter (h)	10	-10		

h = human; [1] data from study U10-2617-01; [2] data from study U10-2610-01
