# Supplementary material for: CCR3 plays a role in murine age-related cognitive changes and T-cell infiltration into the brain
Source: Commun Biol. 2023 Mar 18;6:292. doi: 10.1038/s42003-023-04665-w (PMC10024715; doi:10.1038/s42003-023-04665-w)
Supplement: Supplementary file 2 — Description of Additional Supplementary Files [file 42003_2023_4665_MOESM2_ESM.pdf]

Description of Additional Supplementary Files

File Name: Supplementary Data

Description: The source data that support the main findings of this study.
