# Supplementary material for: CCR3 plays a role in murine age-related cognitive changes and T-cell infiltration into the brain
Source: Commun Biol. 2023 Mar 18;6:292. doi: 10.1038/s42003-023-04665-w (PMC10024715; doi:10.1038/s42003-023-04665-w)
Supplement: Supplementary file 4 — Reporting Summary [file 42003_2023_4665_MOESM4_ESM.pdf]

nature portfolio

Corresponding author(s): Arnaud Teichert

Last updated by author(s): Feb 6, 2023

Reporting Summary

Nature Portfolio wishes to improve the reproducibility of the work that we publish. This form provides structure for consistency and transparency in reporting. For further information on Nature Portfolio policies, see our Editorial Policies and the Editorial Policy Checklist.

Statistics

For all statistical analyses, confirm that the following items are present in the figure legend, table legend, main text, or Methods section.

n/a	Confirmed	
☐	☒	The exact sample size (*n*) for each experimental group/condition, given as a discrete number and unit of measurement
☐	☒	A statement on whether measurements were taken from distinct samples or whether the same sample was measured repeatedly
☐	☒	The statistical test(s) used AND whether they are one- or two-sided<br>*Only common tests should be described solely by name; describe more complex techniques in the Methods section.*
☐	☒	A description of all covariates tested
☐	☒	A description of any assumptions or corrections, such as tests of normality and adjustment for multiple comparisons
☐	☒	A full description of the statistical parameters including central tendency (e.g. means) or other basic estimates (e.g. regression coefficient) AND variation (e.g. standard deviation) or associated estimates of uncertainty (e.g. confidence intervals)
☐	☒	For null hypothesis testing, the test statistic (e.g. *F*, *t*, *r*) with confidence intervals, effect sizes, degrees of freedom and *P* value noted<br>*Give P values as exact values whenever suitable.*
☒	☐	For Bayesian analysis, information on the choice of priors and Markov chain Monte Carlo settings
☒	☐	For hierarchical and complex designs, identification of the appropriate level for tests and full reporting of outcomes
☐	☒	Estimates of effect sizes (e.g. Cohen's *d*, Pearson's *r*), indicating how they were calculated

Our web collection on statistics for biologists contains articles on many of the points above.

Software and code

Policy information about availability of computer code

Data collection	None used.
Data analysis	Seurat, v3. Reference is in the Methods section: Butler et al, 2018.

For manuscripts utilizing custom algorithms or software that are central to the research but not yet described in published literature, software must be made available to editors and reviewers. We strongly encourage code deposition in a community repository (e.g. GitHub). See the Nature Portfolio guidelines for submitting code & software for further information.

Data

Policy information about availability of data

All manuscripts must include a data availability statement. This statement should provide the following information, where applicable:

- Accession codes, unique identifiers, or web links for publicly available datasets
- A description of any restrictions on data availability
- For clinical datasets or third party data, please ensure that the statement adheres to our policy

The data that support the main findings of this study are available in the Supplementary Data files. Data for findings in the Supplementary Information are available from the corresponding author upon reasonable request.

Human research participants

Policy information about studies involving human research participants and Sex and Gender in Research.

Reporting on sex and gender	No human participants in this study.
Population characteristics	None.
Recruitment	None.
Ethics oversight	None.

Note that full information on the approval of the study protocol must also be provided in the manuscript.

Field-specific reporting

Please select the one below that is the best fit for your research. If you are not sure, read the appropriate sections before making your selection.

☒ Life sciences ☐ Behavioural & social sciences ☐ Ecological, evolutionary & environmental sciences

For a reference copy of the document with all sections, see nature.com/documents/nr-reporting-summary-flat.pdf

Life sciences study design

All studies must disclose on these points even when the disclosure is negative.

Sample size	Sample size for all rodent studies were based on published literature with similar assays and techniques, as well as our own pilot studies validating proof-of-concept and reproducibility. Studies with behavioral and cognitive tests utilized sample sizes of n=15 or more per group. Studies with immunohistochemistry utilized a sample size of n=5 or more depending on variability determined from prior studies.
Data exclusions	Individual data points were excluded as outliers when they were greater than two standard deviations from the mean. No prior exclusion criterion were used for rodent studies.
Replication	Each experiment was replicated at least 3 times successfully.
Randomization	Animals were stratified evenly into different treatment groups using a ranking system that utilized weight and locomotor function (distance and velocity).
Blinding	All experiments were blinded: the operators responsible for the experimental procedures and data analysis were blinded and unaware of group allocation throughout the experiments.

Reporting for specific materials, systems and methods

We require information from authors about some types of materials, experimental systems and methods used in many studies. Here, indicate whether each material, system or method listed is relevant to your study. If you are not sure if a list item applies to your research, read the appropriate section before selecting a response.

Materials & experimental systems

n/a	Involved in the study	
☐	☒	Antibodies
☒	☐	Eukaryotic cell lines
☒	☐	Palaeontology and archaeology
☐	☒	Animals and other organisms
☒	☐	Clinical data
☒	☐	Dual use research of concern

Methods

n/a	Involved in the study	
☒	☐	ChIP-seq
☐	☒	Flow cytometry
☒	☐	MRI-based neuroimaging

Antibodies

Antibodies used	All antibodies are listed in the method section. Briefly, we used the following primary antibodies for immunohistochemistry: rat anti-CD3 (555273, BD Biosciences, 1:100), rabbit anti-CD45 (702575, Cell Signaling, 1:200), rabbit anti-Iba1 (016-26721, Wako, 1:1000), rat anti-CD68 (MCA1957, Biorad, 1:1000), rabbit anti-GFAP (Z033429-2, Dako, 1:1000), and guinea-pig anti-TMEM119 (400

004,Synaptic Systems, 1:200). The appropriate fluorescent secondary antibodies (Alexa-488/555/647, Invitrogen, 1:300) were used. We used the following conjugated antibodies for flow cytometry: anti-CD45-FITC (103108, BioLegend, 1:100), anti-CD170-AF 700 (56-1702-82, ThermoFisher, 1:100), anti-CD3-PerCP-efluor710 (46-0032-82, ThermoFisher, 1:50), anti-CCR3 AF647 (FAB1551R, Novus, 1:50), anti-CD4 eFluor 506 (69-0042-82, ThermoFisher, 1:50), anti-CD8 Super Bright 600 (63-0081-82, ThermoFisher, 1:50), anti-Ly-6G (Gr-1) PE-eFluor 610 (61-9668-82, ThermoFisher, 1:50), anti-B220 (CD45RT) PE-Cyanine7 (25-0452-82, ThermoFisher. 1:50), hamster anti-CD11c APC (Clone N418; 117310, BioLegend, 1:100), rat anti-CD8a APC-Cyanine7 (Clone 53-6.7, 100714, Biolegend, 1:100), rat anti-CCR3 PE (Clone 83103, FAB1551P, Novus Biologicals, 1:100), hamster anti-CD4 PE-Cyanine7 (Clone GK1.5; 25-0041-82, eBioscience, 1:100), rat anti-I-A/I-E (MHCII)-BV421 (Clone M5/114.15.2; 107632, BioLegend, 1:100), rat anti-CD19 BV605 (Clone 6D5. 115540, BioLegend, 1:100), rat anti-Ly6g – BV605 (Clone 1A8; 127639, BioLegend, 1:100), and rat anti-CD11b - SB702 (Clone M1/70; 67-0112-82, eBioscience, 1:100).

Validation

Antibodies were previously used and data were published in literature and on manufacturer's websites: Monoclonal rat anti-CD3 (555273, BD Biosciences, 1:100); Miescher GC et al. Immunol Lett. 1989; 23(2):113-118. Monoclonal rabbit anti-CD45 (702575, Cell Signaling, 1:200); Yao Yuan, et al. Nat Commun. 2020.
Monoclonal rabbit anti-Iba1 (016-26721, Wako, 1:1000); Takata K, et al. Front Line Microglia Res. 2020. Monoclonal rat anti-CD68 (MCA1957, Biorad, 1:1000); Garofalo S. et al. J Neurosci. 2017.
Polyclonal guinea-pig anti-TMEM119 (400 004, Synaptic Systems, 1:200); Guldner IH. Cell. 2020. Alexa Flour conjugated donkey anti-mouse/rat/rabbit/goat (Alexa-488/555/647, Invitrogen, 1:300) Flow antibodies:
Monoclonal rat anti-CD45-FITC (103108, BioLegend, 1:100); Podd BS., et al. J. Immunol. 2006. 176:6532
Monoclonal rat anti-CD170-AF 700 (56-1702-82, ThermoFisher, 1:100); Hardy, et al. J Autoimmun. 2018. 92:104-113 Monoclonal rat anti-CD3-PerCP-efluor710 (46-0032-82, ThermoFisher); Schleicher, et al. Cell Rep. 2016. 15(5):1062-1075 Monoclonal rat anti-CCR3 AF647 (FAB1551R, Novus, 1:50); Sullivan, et al. J Leukoc Biol. 2018. 104(1): 11-19
Monoclonal rat anti-CD4 eFluor 506 (69-0042-82, ThermoFisher, 1:50); Gao, et al. Front Neurol. 2017. 8:281
Monoclonal rat anti-CD8 Super Bright 600 (63-0081-82, ThermoFisher, 1:50); Gao, et al. Front Neurol. 2017. 8:281
Monoclonal rat anti-Ly-6G (Gr-1) PE-eFluor 610 (61-9668-82, ThermoFisher, 1:50), Cai, et al. Cell Res. 2016. 26(8):886-900
Monoclonal rat anti-B220 (CD45RT) PE-Cyanine7 (25-0452-82, ThermoFisher. 1:50). Perez-Chacon, et al. Front Immunol. 2019. 11;9:3114
Monoclonal hamster anti-CD11c - APC (Clone N418; 117310, BioLegend, 1:100) Monoclonal rat anti-CD8a – APC/Cyanine7 (Clone 53-6.7; 100714 BioLegend, 1:100) Monoclonal rat anti-CCR3 – PE (Clone 83103; FAB1551P, Novus Biologicals, 1:100)
Monoclonal hamster anti-CD4 - PE-Cyanine7 _Clone GK1.5; 25-0041-82, eBioscience, 1:100) Monoclonal rat anti-I-A/I-E (MHCII)-BV421 (Clone M5/114.15.2; 107632, BioLegend, 1:100) Monoclonal rat anti-CD19 - BV605 (Clone 6D5. 115540, BioLegend, 1:100)
Monoclonal rat anti-Ly6g – BV605 (Clone 1A8; 127639, BioLegend, 1:100) Monoclonal rat anti-CD11b - SB702 (Clone M1/70; 67-0112-82, eBioscience, 1:100)

Animals and other research organisms

Policy information about studies involving animals; ARRIVE guidelines recommended for reporting animal research, and Sex and Gender in Research

Laboratory animals

All mice were individually housed under specific pathogen-free conditions under a 12-hour light, 12-hour dark cycle. All animal handling and use was in accordance with Institutional Animal Care and Use Committee using the National Institute of Health guidelines. Mice were weighed upon arrival and weekly thereafter until study completion. Animals were either single housed or group housed in cages of 5 with a unique identification number. All animals used were C57BL/6 mice purchased from Jackson Laboratories. Young mice were 2-3 months old and aged mice were 20-24 months old.

Wild animals

This study did not involve wild animals.

Reporting on sex

All mice used were male, with the exception of MOG-induced T cell model, which included males and females (Figure 5). These experiments with both sexes were not statistically different from each other and thus were combined and reported as such. Males and females were approximately evenly numbered in these experiments.

Field-collected samples

This study did not involve samples collected from the field.

Ethics oversight

All procedures were approved by the Institutional Animal Care and Use Committee at Alkahest, Inc. following the guidelines set forth by AAALAC and The Guide.

Note that full information on the approval of the study protocol must also be provided in the manuscript.

Flow Cytometry

Plots

Confirm that:

☒ The axis labels state the marker and fluorochrome used (e.g. CD4-FITC).

☒ The axis scales are clearly visible. Include numbers along axes only for bottom left plot of group (a 'group' is an analysis of identical markers).

☒ All plots are contour plots with outliers or pseudocolor plots.

☒ A numerical value for number of cells or percentage (with statistics) is provided.

Methodology

Sample preparation	Blood was collected via cardiac puncture into an EDTA-coated syringe and 100 µl aliquoted into flow cytometry tube at room temperature. All samples were processed for flow cytometry at the same time. Red blood cells were lysed with 1X lyse/fix buffer (558049, BD Biosciences) for 10 minutes at room temperature (RT) followed by a spin at 500g for 8 minutes RT. The supernatant was discarded, cells were washed in HBSS followed by another spin at 500g for 8 minutes RT. The cell pellet was resuspended in 100 µl of stain buffer (554656, BD Biosciences) and incubated at RT in antibody solution for 30 minutes in the dark. Cells were then washed in stain buffer and the final pellet resuspended in 300 µl of stain buffer.
Instrument	Data was acquired on an Attune NxT Flow Cytometer (ThermoFisher, A24858).
Software	Data was analyzed with FlowJo Analysis Software (V10.6).
Cell population abundance	Cell Abundance for subpopulations of cells were normalized to CD45 expression in the sample. Cell sample purity was maintained by keeping whole blood samples separated and processed within 1 hour of collection to minimize the possibilities of contaminations.
Gating strategy	Gating strategy used in all experiments is illustrated in Supplementary Figure 3. Briefly, an initial FSC vs SSC plot was used to gate out low FSC cells (debris) followed by a FSC-A vs FSC-H plot to gate out doublets and further debris. Positive antibody signal were defined using Fluorescent Minus One (FMO) samples, FSC, and SSC to distinguish positive signal from negative signal within each fluorochrome. The FMO was used in all experiments if essential to the gating or only during the pilot experiments when not essential for the gating strategy. Such as when the FMO would be redundant with the use of FSC/SSC.

☒ Tick this box to confirm that a figure exemplifying the gating strategy is provided in the Supplementary Information.
